# Safari in the RNA world: a special issue focused on RNA biogenesis, functions, and technologies

**DOI:** 10.3724/abbs.2024234

**Published:** 2025-01-25

**Authors:** Ya-Nan Chang, Hong Cheng

**Affiliations:** 1 Key Laboratory of Systems Health Science of Zhejiang Province School of Life Science Hangzhou Institute for Advanced Study University of Chinese Academy of Sciences Hangzhou 310024 China; 2 Key Laboratory of RNA Innovation Science and Engineering Shanghai Key Laboratory of Molecular Andrology Shanghai Institute of Biochemistry and Cell Biology Center for Excellence in Molecular Cell Science Chinese Academy of Sciences University of Chinese Academy of Sciences Shanghai 200031 China

RNA is one of the most essential biopolymers in cells. According to the central dogma, messenger RNAs (mRNAs) transmit genetic information from DNA to proteins through a complex process, facilitated by key non-coding RNAs (ncRNAs) such as ribosomal RNAs (rRNAs) and transfer RNAs (tRNAs). In addition to these essential RNAs, a wide variety of ncRNAs have been discovered, each playing key roles in gene regulation and contributing to the complexity of the RNA landscape.

All RNAs undergo intricate processing and modifications before maturation and transport to their respective cellular compartments, where they perform their functions. Regulation of these processes often results in the generation of multiple isoforms from a single gene, further diversifying the RNA landscape. Understanding the mechanisms of RNA biogenesis and the functional roles of RNAs in both physiological and pathological contexts is essential for unraveling how cells respond to developmental and environmental cues, with profound implications for biomedicine. This special issue features 12 expert reviews in RNA research, each offering a comprehensive summary of the latest advances in RNA biology from their respective perspectives.

Alternative splicing is a key post-transcriptional process that expands the functional proteome in eukaryotes. Bao *et al*. [1] discuss how RNA structures regulate alternative splicing and the impact of aberrant splicing on disease phenotypes, along with strategies to correct these defects. Zhang *et al*. [2] highlight the crosstalk between splicing and 3′-end processing, which involves selective polyadenylation site usage to produce isoforms with varying 3′ UTR lengths and different open reading frame sequences. Additionally, they explore mechanisms behind this crosstalk and how long-read sequencing can help identify these regulatory interactions, offering potential for improved disease diagnosis and treatment.

RNA editing and modification represent frontier areas in RNA research. The same RNA molecule can have different fates and functions depending on its modifications. In a review by Wen *et al*. [3], the authors explore the regulatory roles and biological functions of various RNA modifications in mammalian development, with a particular focus on reproduction and embryonic development. *N* ^6^-methyladenosine (m ^6^A) is the most prevalent epigenetic modification in eukaryotic mRNAs and plays a key role in regulating gene expression by influencing multiple aspects of mRNA metabolism. A review by Deng *et al*. [4] provides a structural perspective on recent advances in the properties of m ^6^A writers, focusing on how they recognize RNA substrates and mediate selective m ^6^A modifications. Relatedly, Mao *et al*. [5] discusses RNA editing, particularly A-to-I and C-to-U editing of tRNAs by the ADAT protein family, and addresses the association of mutations in these processes with disease.

After processing and modification, mature mRNAs are exported from the nucleus to the cytoplasm. Chen *et al*. [6] outlines the current understanding of mRNA export in higher eukaryotic cells, emphasizing its role in gene expression regulation and how it integrates with other processes to ensure precise genetic information transmission. Recent advances in RNA proximity labeling and fluorogenic RNA aptamers have revolutionized our understanding of RNA transport and localization. Liu *et al*. [7] introduces various tools for RNA proximity labeling and explores their use in studying the spatiotemporal regulation of RNA. Fluorogenic RNA aptamers have proven to be powerful tools for observing the transport and localization of individual RNAs. In a review by Song *et al* . [8], the structural investigation of these aptamers is summarized, highlighting how structural insights can guide the optimization of RNA visualization techniques.

Small regulatory RNAs, such as miRNAs and piRNAs, play crucial roles in eukaryotes by influencing gene regulation, developmental timing, antiviral defense, and genome integrity through RNA interference (RNAi). Feng *et al*. [9] focuses on the biological roles of the RNAi pathway and provides an overview of its applications in biomedical research, agriculture, and therapeutics. In plants, transitive small interfering RNAs (siRNAs), generated through RNA-dependent RNA polymerases (RDRs), can amplify and spread silencing signals to additional transcripts. A review by Tan *et al*. [10] discusses the biogenesis and biological functions of transitive siRNAs. Relatedly, a review by Lv *et al*. [11] highlights recent discoveries of PIWI-piRNA functions in oogenesis and embryogenesis in golden hamsters and explores new directions for piRNA research.

Finally, circular RNAs (circRNAs) have emerged as stable and conserved molecules primarily derived from back-splicing of pre-mRNAs. Zhang *et al*. [12] briefly discusses the non-coding and coding functions of circRNAs in cells, as well as methods for their *in vitro* synthesis and recent advances in their biomedicine applications.

In conclusion, this special issue enhances our understanding of the complex RNA world. By introducing diverse RNA types and their intricate regulatory processes, it sheds light on the complexity of RNA biogenesis. It also highlights how new technologies are advancing our understanding of RNA processing, modification, and their interconnections with RNA transport and localization. These RNA species and regulatory mechanisms play critical roles in various physiological processes, and when disrupted, can lead to a wide range of diseases. Unsurprisingly, modulation of RNA processing and the application of different RNA types have emerged as promising therapeutic approaches. The reviews in this issue have the potential to set new directions and provide insightful hypotheses in RNA research, paving the way for future scientific breakthroughs.
